# Current opinions on the present and future use of functional near-infrared spectroscopy in psychiatry

**DOI:** 10.1117/1.NPh.10.1.013505

**Published:** 2023-02-07

**Authors:** Rihui Li, Hadi Hosseini, Manish Saggar, Stephanie Christina Balters, Allan L. Reiss

**Affiliations:** a Stanford University, Center for Interdisciplinary Brain Sciences Research, Department of Psychiatry and Behavioral Sciences, Stanford, California, United States; b Stanford University, Department of Radiology and Pediatrics, Stanford, California, United States; c Stanford University, Department of Pediatrics, Stanford, California, United States

**Keywords:** functional near-infrared spectroscopy, psychiatry, brain function

## Abstract

Functional near-infrared spectroscopy (fNIRS) is an optical imaging technique for assessing human brain activity by noninvasively measuring the fluctuation of cerebral oxygenated- and deoxygenated-hemoglobin concentrations associated with neuronal activity. Owing to its superior mobility, low cost, and good tolerance for motion, the past few decades have witnessed a rapid increase in the research and clinical use of fNIRS in a variety of psychiatric disorders. In this perspective article, we first briefly summarize the state-of-the-art concerning fNIRS research in psychiatry. In particular, we highlight the diverse applications of fNIRS in psychiatric research, the advanced development of fNIRS instruments, and novel fNIRS study designs for exploring brain activity associated with psychiatric disorders. We then discuss some of the open challenges and share our perspectives on the future of fNIRS in psychiatric research and clinical practice. We conclude that fNIRS holds promise for becoming a useful tool in clinical psychiatric settings with respect to developing closed-loop systems and improving individualized treatments and diagnostics.

## 1 Introduction

Psychiatric disorders have been cited as a global public health issue due to the high growth rate of diagnosed individuals over the past decades.^1^ At present, the diagnosis of most psychiatric disorders is based largely on associated descriptive symptoms and signs, lacking objective biomarkers. Patients who are unable to accurately identify and express their symptoms may be difficult to identify and diagnose. These circumstances may be even more common in children, for whom case symptoms may need to be endorsed by parents or teachers (and reports are sometimes inconsistent). This has prevented many individuals with psychiatric symptoms from receiving appropriate treatment and, thus, experiencing better outcomes. Over the past decades, the development of advanced neuroimaging technologies, such as functional magnetic resonance imaging (fMRI), positron emission tomography (PET), and electroencephalography (EEG), have enabled researchers to explore a wide range of brain functions with more objective measures and expand our current understanding of the underlying mechanisms associated with a variety of psychiatric conditions.^2^^–^^10^

Functional near-infrared spectroscopy (fNIRS) is a noninvasive optical imaging method for measuring and imaging the functional hemodynamic response to brain activity.^11^ In general, fNIRS uses near-infrared light sources with wavelengths between 650 and 1000 nm that can propagate several centimeters through the scalp and skull and spectroscopically interrogate the fluctuated concentrations of oxygenated and deoxygenated hemoglobin, a metabolic process corresponding to neuronal response within the brain.^12^^,^^13^ The past three decades have seen rapid growth of fNIRS as a valuable tool for studying normal brain function and its alteration in diseases.^14^^–^^18^ Technically, fNIRS has several advantages over other commonly used functional neuroimaging techniques; it is portable and resilient to motion artifacts, and it offers higher spatial resolution and temporal resolution compared with EEG and fMRI, respectively.^19^^–^^22^ The flexible applicability and high ecological validity of fNIRS have made it particularly suitable for probing the brain activity of participants, including pediatric populations, who may fear stressful environments (e.g., MRI scanners) or display motor restlessness and anxiety symptoms [e.g., autism spectrum disorder (ASD)]. In the present opinion article, we briefly summarize state-of-the-art fNIRS research in psychiatry and share our perspectives on its future applicability in psychiatric research and clinical practice.

## 2 Current Status of FNIRS in Psychiatry Studies

### 2.1 Applications of fNIRS in Psychiatric Disorders

**Fig. 1:**
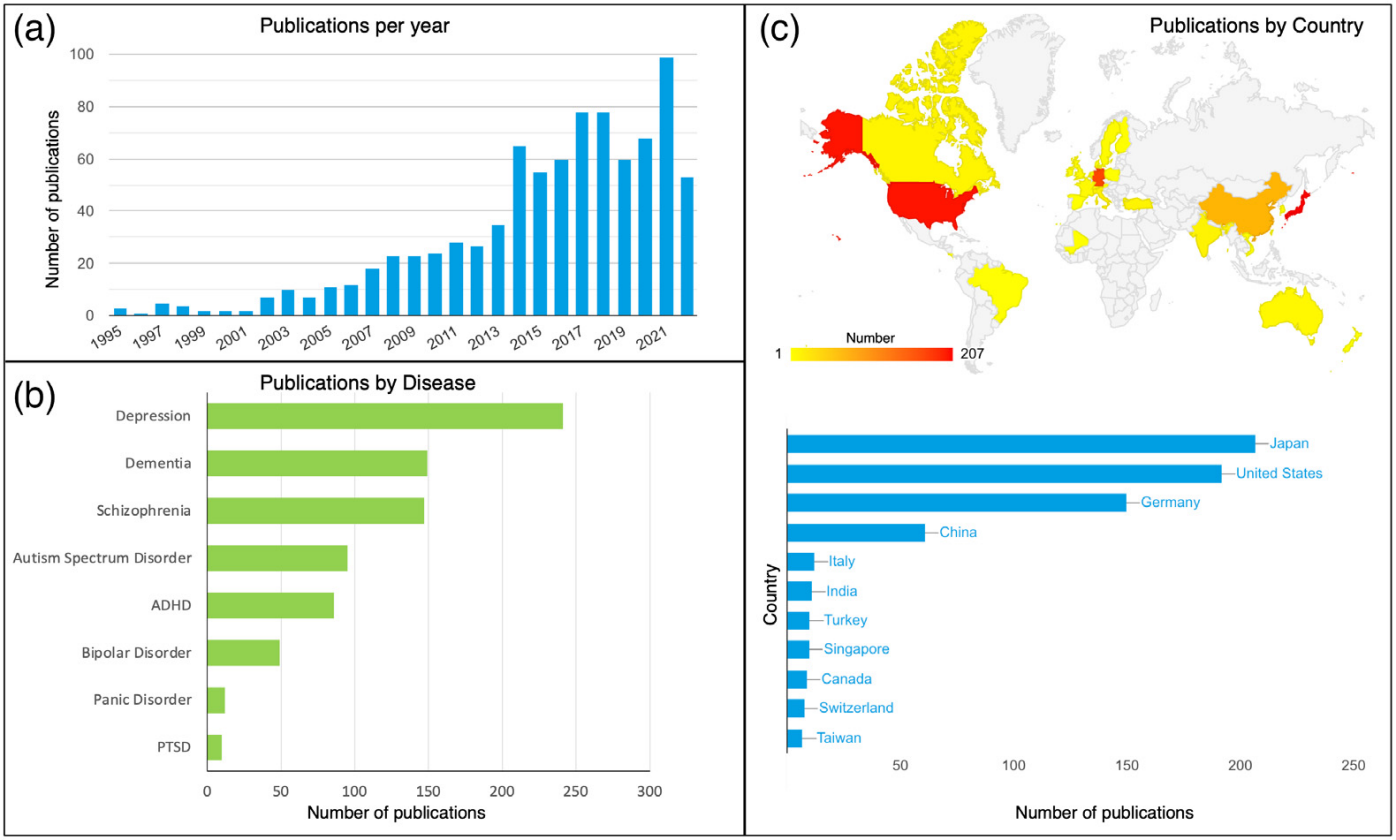
Review of the literature. (a) The annual growth of publications in fNIRS-related psychiatric research. (b) The distribution of major psychiatric disorders primarily studied in previous fNIRS publications. (c) The distribution of relevant fNIRS publications by country. These statistics were obtained from a scholar search website^43^ and Web of Science search with the keywords (“fNIRS” or “NIRS”) and (“psychiatry” or “psychiatric”).

Relative to other functional imaging techniques, fNIRS is advantageous for patients with psychiatric disorders as it does not require subjects to be completely still, asleep, or sedated. Functional NIRS also allows individuals undergoing imaging to interact freely with their environment.^23^^–^^26^ Since the first fNIRS study on patients with schizophrenia was published in 1994,^27^ fNIRS has increasingly been used to explore a number of psychiatric disorders, including schizophrenia,^28^^,^^29^ depression,^30^^,^^31^ bipolar disorder,^32^^,^^33^ panic disorder,^34^ obsessive-compulsive disorder,^35^ Alzheimer’s disease,^36^^,^^37^ ASD,^38^^,^^39^ attention-deficit hyperactivity disorder (ADHD),^40^^,^^41^ and posttraumatic stress disorder (PTSD).^42^ Figure 1 illustrates the growth of fNIRS publications in psychiatric disorders for the past 30 years. Overall, the number of fNIRS publications focused on psychiatric disorders has increased rapidly over the past 10 years [Fig. 1(a)], possibly owing to the technological innovations in hardware instruments (e.g., time-domain NIRS^44^ and wearable NIRS^45^^,^^46^), signal processing (e.g., artifact removal^47^^,^^48^ and real-time processing^49^), and paradigm design (e.g., hyperscanning^24^^,^^25^). As shown in Fig. 1(b), the mainstream fNIRS literature covers a variety of psychiatric disorders, with depression, dementia, schizophrenia, ASD, and ADHD accounting for the top five conditions of interest. In terms of regional distribution, Asia, America, and Europe, as represented by Japan and China, the United States, and Germany, respectively, have made significant contributions to bringing fNIRS methods into psychiatric research [Fig. 1(c)].

Regarding the role of fNIRS in psychiatric research, the focus of the literature is to ascertain differences between patients with various types of psychiatric disorders and healthy controls, with the aim of characterizing neural abnormalities potentially underlying the pathogenesis of a condition. For example, many studies reported abnormal brain activation and reduced hemodynamic responses in the frontal cortex of patients with schizophrenia when compared with the healthy control group,^50^^–^^52^ suggesting frontal cortical abnormalities in this psychiatric disorder. Aberrant cortical activation patterns in the frontal cortex were also widely reported using fNIRS in affective disorders including major depressive disorder (MDD) and bipolar disorder,^32^^,^^33^^,^^53^^,^^54^ though different tasks may induce a wide range of condition-specific abnormal frontal activation. Further investigations also included multiple patient groups with different psychiatric disorders that might share partially overlapping phenotypes, such as MDD and bipolar depression,^32^ MDD, and schizophrenia,^55^ with the aim of delineating disease-specific neural biomarkers among these disorders.

In addition to focusing on identifying brain variations in psychiatric patients relative to healthy controls, other fNIRS studies have focused on elucidating the developmental trajectory of specific disorders. These studies generally conducted cross-sectional investigations of the different stages of neurodegenerative diseases such as Alzheimer’s disease (AD), with the focus on elucidating neural biomarkers of disease progression (e.g., preclinical, mild, moderate stage of AD).^37^^,^^56^ Other studies examined longitudinal change within the same patient cohort to establish neuroimaging indicators that might contribute to the detection of symptom severity over time.^57^^,^^58^ On the other hand, the association between brain hemodynamic response alterations and treatment outcomes following intervention was also explored. Numerous lines of evidence have shown that longitudinal changes in hemodynamic response measured by fNIRS were significantly associated with the treatment outcomes in patients with MDD after different therapies.^59^^–^^61^ Overall, these studies support the feasibility of utilizing fNIRS to assess cross-sectional and longitudinal neural signatures related to psychiatric disorders.

Behavioral rating scales are subject to low sensitivity and specificity and high inter-rater variation for assessing cognitive-behavioral symptoms of psychiatric patients. Recently, emerging evidence has shown that fNIRS-derived neuro-biomarkers might be used as intermediate outcome measures for assessing pharmacological or intervention effects in clinical trials of psychiatric disorders. Several studies applied fNIRS to assess hemodynamic response patterns induced either by methylphenidate (MPH) or by olfactory stimulation in patients with ADHD.^62^^–^^64^ Results showed that hemodynamic responses may be potentially related to neural modulation from these treatments. Apart from pharmacological effects, fNIRS has been widely used to examine the effects of different neuromodulation and nonpharmacological treatments on patients with depression and PTSD, such as transcranial magnetic stimulation (TMS), repetitive TMS (rTMS), and transcranial electrical stimulation (TES).^65^^–^^69^ These studies indicate that neuromodulation techniques that improve psychiatric symptoms can be reflected by changes in cortical hemodynamic responses.

### 2.2 Advanced fNIRS Instrumentation and Paradigms in Psychiatric Studies

**Fig. 2:**
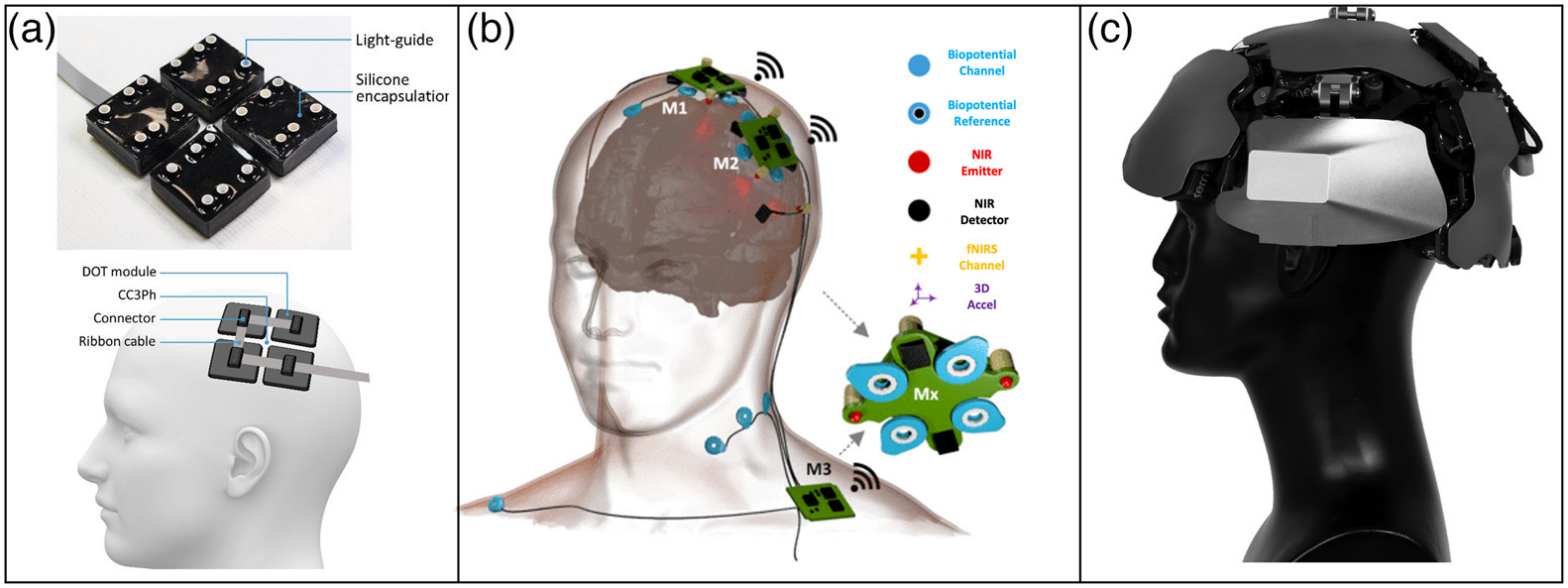
Mobile, modular, and wearable fNIRS systems. (a) A wearable fNIRS device consisting of DOT modules for modular design. Adapted with permission from Ref. 45. (b) A device that allows for simultaneous recording of fNIRS, EEG, and other physiological signals. Adapted with permission from Ref. 72. (c) A time-domain fNIRS device with a miniaturized design and whole-head coverage. Adapted with permission from Ref. 73.

The mobility and flexibility of fNIRS systems are particularly well-suited for psychiatric studies that involve patients with affective disorders, motor restlessness, and anxiety symptoms (e.g., ASD, ADHD, and anxiety disorder). However, typical fNIRS systems used in early psychiatric studies were accompanied by methodological constraints that hindered broader applications to the field. These constraints included poor spatial resolution, low signal-to-noise ratio, and nonportable structures such as long and heavy probe sets and large control units.^12^^,^^17^^,^^23^ Over the past 10 years, fNIRS systems have rapidly advanced toward modular, wireless, and wearable designs that increase the potential scope of psychiatric applications.^70^^,^^71^ For instance, a highly modular, scalable, and wearable diffuse optical tomography (DOT) system, a special type of fNIRS device with enhanced spatial resolution, was developed to enable investigations of brain activity associated with natural behaviors in ecologically valid settings^45^ [Fig. 2(a)]. In addition, a mobile and modular hybrid device (EEG + fNIRS) was developed to allow for a simultaneous multimodal biosignal recording.^72^ This design is one example that sets the stage for future fNIRS systems that incorporate multiple neural and physiological sensors in a modular design [Fig. 2(b)]. Recently, modular and wearable time-domain fNIRS systems have also been developed (e.g., Ref. 73). These systems can be built with miniaturized laser drivers, custom integrated circuits, and specialized detectors that allow for dense channel coverage over the entire head [Fig. 2(c)].

**Fig. 3:**
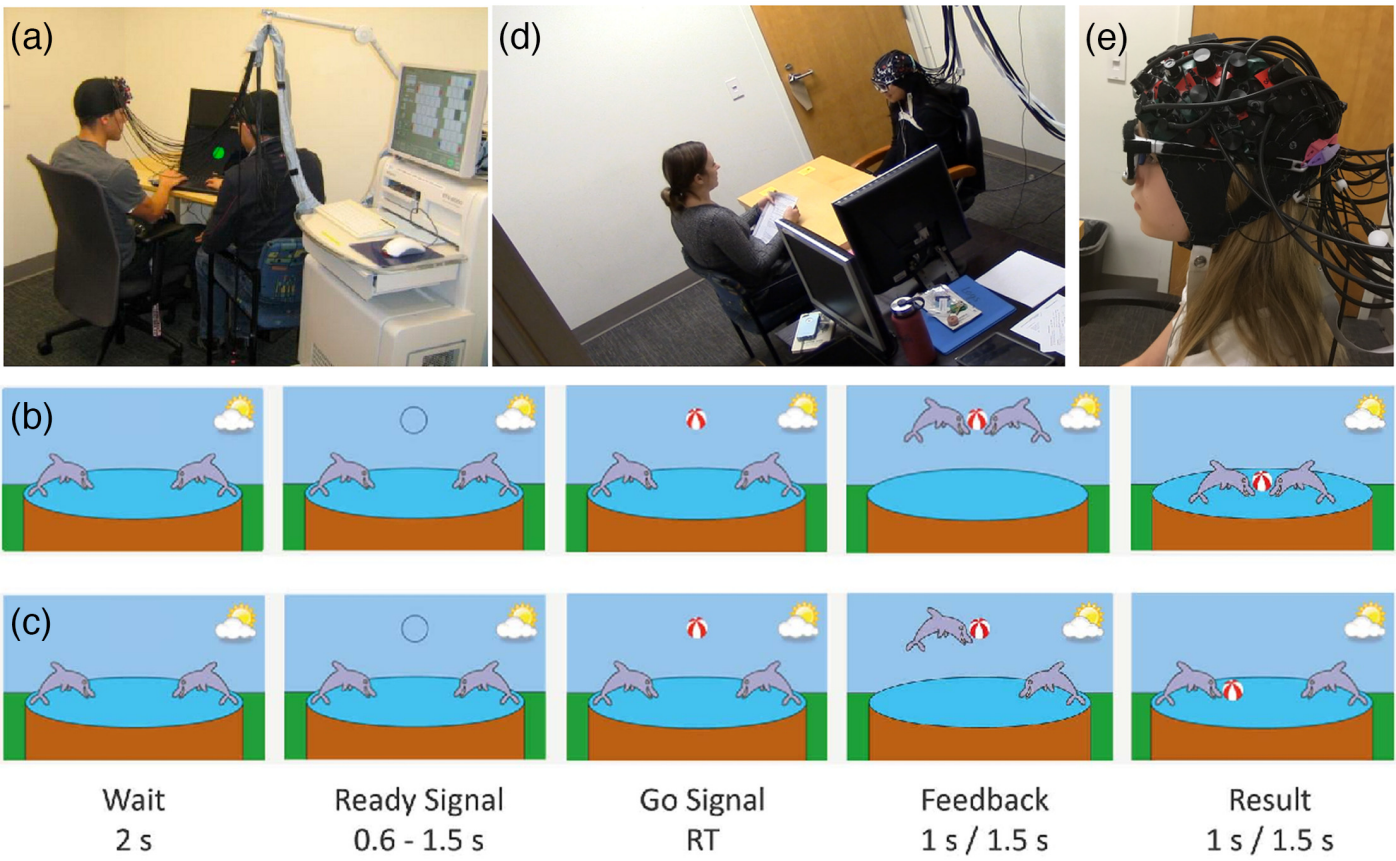
Applications of fNIRS in social interaction. (a) A representative hyperscanning fNIRS experiment measuring inter-brain synchronization during social cooperation and competition. Adapted with permission from Ref. 24. (b) and (c) A typical hyperscanning game was used to study inter-brain synchronization between parent and child with ASD during a cooperation game and a competitive game. Adapted with permission from Ref. 74. (d) and (e) Simultaneous fNIRS and eye tracking recording in girls with fragile X syndrome during a natural conversation. Adapted with permission from Ref. 75.

Taking full advantage of the high mobility of fNIRS systems, researchers have investigated atypical brain dynamics of psychiatric disorders in more unconstrained and natural environments. One of the most exciting applications of fNIRS is hyperscanning, in which brain activities are recorded from two or more participants simultaneously, permitting a direct investigation of quantitative links between two or more brains during interpersonal interaction [Fig. 3(a)].^76^^,^^77^ Since the first fNIRS-based hyperscanning study published in 2012,^24^ this approach has been rapidly applied to studying neural dynamics during social interaction among healthy groups (e.g., teacher-student and mother-child),^78^^–^^81^ as well as patients with primary or secondary social dysfunction, such as ASD^74^^,^^82^^,^^83^ and depression,^14^^,^^84^ respectively. A typical paradigm used to study interbrain synchrony in parent-child dyads is shown in Figs. 3(b) and 3(c). In addition to hyperscanning, fNIRS is utilized in psychiatric studies to investigate a wide range of cognitive-behavioral characteristics in individuals with psychiatric disorders using real-world tasks. Novel paradigms include face recognition,^42^ imitation action,^85^^,^^86^ eye gaze contact,^87^^,^^88^ verbal fluency,^89^ delayed working memory,^90^ assessment of brain development in remote geographical or low-resource areas,^91^^,^^92^ and real-time neurofeedback (NF).^93^^,^^94^ It is also important to note that fNIRS can be used jointly with other bio-behavioral measurements such as eye-tracking devices. As shown in Figs. 3(d) and 3(e), we conducted simultaneous fNIRS and eye tracking recording in girls with fragile X syndrome (FXS) during a natural conversation to show aberrant neural response and eye gaze patterns associated with this genetic condition.^75^ All of the aforementioned studies highlight the flexibility of fNIRS in studying the cognitive-behavioral characteristics of patients with neuropsychiatric and neurodevelopmental disorders.

## 3 Perspective of fNIRS in Future Psychiatry Research

### 3.1 Development of fNIRS Instrumentation and Multimodal Integration

The recent development of wireless and wearable fNIRS systems allows for broader research applications in psychiatry.^45^^,^^46^^,^^71^^,^^72^ However, enhancement of fNIRS instruments should be considered for applying fNIRS to the future study of populations of patients with psychiatric disorders. Specifically, patients with psychiatric disorders such as ASD, bipolar disorder, and ADHD, often display motor restlessness, anxiety, or hyperarousal symptoms that require specific consideration when using fNIRS. Key factors in instrument design to be considered should include (1) user-friendly materials for comfort optode contact, (2) lightweight and low-burden design for enhanced measurement experience and experimental compliance, and (3) advanced signal processing algorithms and noise reduction capacity for robust system performance during long-duration, real-world study (e.g., field studies in low-resource areas). We envision that these challenges will be addressed in the next several years. In particular, highly wearable fNIRS systems could benefit from the steady progress in NIRS light source and sensor development. Large-size laser sources are being replaced by cheaper and smaller light emitters diode sources with comparable performance and high flexibility.^12^^,^^95^ Single-photon avalanche diode and silicon photomultipliers sensors are gaining more attention due to their superior sensitivity compared with the conventional avalanche photodiodes and regular silicon photodiodes.^96^ In addition, the optimization of the circuit designs associated with miniaturized, high-performance electronics and the maturation of artifact removal algorithms should further increase the mobility of systems and expand the application scenarios of fNIRS in psychiatric studies.^21^^,^^72^^,^^95^^–^^97^

Simultaneous multimodal data recording, including brain, physiological, and behavioral information, is becoming more common and important for a comprehensive understanding of psychiatric disorders. For instance, concurrent imaging of fNIRS and EEG has provided high spatiotemporal information for investigating brain activity.^20^^,^^98^^–^^102^ Concurrent fNIRS and eye tracking measurement has been adopted to investigate aberrant neural response and associated eye movements in children with genetic risk factors for ASD (e.g., FXS^75^). Physiological or auxiliary signals, such as blood pressure, respiration, and head movement, have been proven to greatly improve the filtering of physiological interference and motion artifacts during the fNIRS signal processing.^103^^–^^106^ In light of these technical and methodological advances, a clear focus for future fNIRS instrument development with respect to applications to psychiatry should be on wearable fNIRS systems that can be effectively integrated with a variety of other sensors and modalities such as EEG, eye tracking devices, physiology modules (e.g., heart rate and skin conductivity), accelerometers, and virtual reality devices. We expect a highly portable and multifunctional fNIRS system will pave the way for new and highly innovative psychiatric studies over the next few years, particularly in the domains of real-world settings.

### 3.2 fNIRS-based Closed-loop Neurofeedback and Treatment

The mobility of wearable fNIRS allows for investigating neural signatures related to the assessment of multidomain cognitive-behavioral functions such as social skills, problem-solving, and emotion processing. However, these investigations are still in the early stages of applying fNIRS in psychiatric research, yet there is no doubt that the next few years will see novel approaches for using this technique. Here we emphasize the potentially important role of fNIRS in real-time NF training and clinical intervention in future research. NF is a specific form of biofeedback that provides users with real-time feedback about their brain activity, thus enabling users to regulate their own brain activity with the aim of improving the target neurocognitive function putatively underlying, e.g., psychiatric symptoms. It has been shown that combined cognitive training and fNIRS-based NF may enhance the executive function of healthy adults after a relatively short period of training.^90^ For example, a pilot study showed that fNIRS-based NF training enhanced therapeutic effects in children with ASD compared with patients who received sham NF training.^93^ For patients with ADHD, several studies showed that fNIRS-NF training resulted in reduced ADHD symptoms comparable to other treatments (i.e., EEG-NF, electromyography-feedback, and medication).^107^^–^^109^ Though none of these approaches has been widely applied in clinical care and treatment, these findings highlight a potential roadmap for developing fNIRS-based NF protocols for treating patients with psychiatric disorders in the future. Such fNIRS-based NF protocols could also enable individuals with psychiatric disorders to receive flexible and independent rehabilitative training at home, perhaps combined with real-time app-based symptom monitoring.

In addition to being used as a tool for computational NF training, an important future application is integrating fNIRS with neural treatments (e.g., neuromodulation) in a closed-loop intervention design. Over the past 10 years, a growing number of studies have utilized fNIRS to explore the brain response of psychiatric patients during or after neuromodulation treatments including TMS and TES.^69^^,^^110^^–^^113^ Together with the trend toward increasing mobility of fNIRS, these studies have given rise to the development of a novel and individualized multimodal intervention approach for psychiatric treatments. Specifically, the fNIRS measurement could serve as a key component of a closed-loop system that monitors brain response in real time and adaptively adjusts stimulation parameters (e.g., intensity and location) or treatment setting (e.g., drug delivery) in a dynamic manner, thus enhancing treatment outcomes for patients. We foresee that this kind of fNIRS-guided closed-loop intervention system may be particularly suitable for regulating psychiatric disorders that manifest symptoms of repetitive, recursive thoughts (e.g., rumination) or behaviors, such as that observed in obsessive-compulsive disorder and other anxiety disorders.

The present and future of fNIRS applications in psychiatry are promising, but there remain several challenges to be addressed before additional, substantive progress can be achieved and applied to the clinical setting. First, both fNIRS-based NF and real-time brain activity monitoring require reliable and real-time signal processing to provide instant feedback. Yet, due to the inherent prolonged delay of hemodynamic response and low signal-to-noise ratio, the majority of conventional fNIRS analyses have primarily relied on offline analysis and multiple trials, neither of which are computationally efficient or technically feasible for real-time analysis. Future applications will also require advanced approaches to remove signal artifact contamination in real time, as well as single-trial signal processing. Both will enhance the real-time brain assessment.^114^^,^^115^ Moreover, the mainstream literature studying brain dynamics associated with specific tasks or neural treatment has focused on group-level analysis that does not take the individual subject variation into consideration. To achieve individualized treatment, it is critical to identify subject-specific neural biomarkers and target brain regions of interest for accurate monitoring of brain activity, thus facilitating the individualization of treatment protocols to obtain enhanced efficiency for individual patients.

### 3.3 Diagnostic Efficacy of fNIRS in Psychiatric Disorders

Another important future perspective for fNIRS applications to psychiatry pertains to diagnostic precision and identifying valid subgroups. Although fNIRS has been widely used to characterize alterations in cerebral oxygenation related to various (phenomenologically defined) psychiatric disorders, its efficacy in screening disease-specific neurobiological signatures is rarely explored, particularly with respect to the sensitivity and specificity of fNIRS measurement in addressing the heterogeneity of psychiatric disorders.

A typical challenge in diagnosing psychiatric disorders is that patients with different risk factors and neurobiological mechanisms may exhibit similar symptoms that lead to heterogeneity in well-accepted psychiatric diagnostic categories. For instance, a broad range of independent genetic influences, such as FXS, increases the risk for ASD.^116^ Although FXS is known to have a clear genetic-biological etiology, many individuals with FXS also manifest cognitive-behavioral symptoms similar to individuals with (nonfragile X) ASD diagnoses.^117^^–^^119^ Therefore, it is becoming increasingly important to identify fNIRS-based biomarkers that are uniquely associated with different neurobiological risk factors for psychiatric disorders, and consequently, to enhance the possibility of measuring the response to syndrome-specific interventions.^75^ To address this issue, we recommend that, in addition to recruiting healthy controls as a contrast group for fNIRS studies, future investigations should recruit patients with comparable symptoms but different diagnoses as comparison groups to elucidate psychiatric biomarkers for the target disease.

Another challenge of using fNIRS as a diagnostic tool for psychiatric disorders is the relatively poor spatial resolution and penetration depth of fNIRS compared with fMRI. These limitations constrain the sensitivity of fNIRS in detecting spatially subtle neural signatures at cortical or subcortical areas that might be critical in disease diagnosis (e.g., anxiety or depressive disorders). Promisingly, high-density DOT systems have demonstrated precise mapping of brain function with fNIRS, pushing its spatial resolution close to that of fMRI.^120^ However, the additional reduction of weight and the ergonomics of such systems still need to be addressed. We envision that portable and fiberless high-density fNIRS devices, including both tomography and topography designs, will be developed and optimized in the upcoming years to reconcile the need for high spatial mapping in psychiatric studies. The effort to tackle the depth limitation of the superficial fNIRS signal has also been made. Through computational methods, several studies have shown that fNIRS signals measured from the cortex can be used to infer fMRI signals measured from deep-brain areas that are critically linked to the pathophysiology of psychiatric disorders, such as the insular cortex, amygdala, and hippocampus.^121^^,^^122^ These findings provide indirect but promising solutions to extend the detectability of fNIRS without sacrificing its cost-effectiveness and portability. To move forward, more studies are required to evaluate how sensitive the fNIRS signal could infer the neural alterations in subcortical areas as well as the abnormal cortical-subcortical connections in patients with different psychiatric disorders.

Integration of multidimensional disease-linked information could also be an effective solution for enhancing the diagnostic efficacy of fNIRS in psychiatric disorders. As noted in Sec. 3.1, multimodal systems integrating portable fNIRS and other neuroimaging techniques, physiological, and behavioral measurements (e.g., EEG, eye tracking, and skin conductance) allow for a simultaneous recording of brain-physiology-behavior markers for psychiatric disorders. We expect that future studies in the field will propose advanced algorithms, most likely based on machine learning techniques, to fuse such multimodal information for more reliable diagnosis and even early prediction of psychiatric disorders.

## 4 Conclusion

The mobility, low cost, and relative resilience to motion artifacts of fNIRS have marked this technology as one of the most promising tools for assessing human brain activity. In the field of psychiatry, we have seen rapid growth in the use of fNIRS for understanding neural mechanisms of various psychiatric disorders and in providing preliminary evidence for refining the treatment of persons with these disorders. However, there remain significant challenges to the wide application of fNIRS to both clinical and research settings, with respect to both instrumentation and signal processing. In particular, the mobility and robustness of fNIRS systems will have to be further advanced together with enhanced spatial resolution and depth to achieve improvements in signal quality and sensitivity. Novel paradigms and new algorithms for single-trial signal processing will be needed to facilitate the routine use of real-time fNIRS NF training and intervention in clinical practice. The integration of multi-dimensional information (e.g., EEG, eye tracking, and heart rate) and artificial intelligence will be invaluable for enabling effective personalized monitoring, diagnosis, and treatment for patients with psychiatric disorders. Finally, all of these improvements should be validated in larger clinical populations with standardized paradigm protocols and data analysis pipelines to ensure sufficient reproducibility and reliability for the clinical applications of fNIRS in psychiatry.
